# Comparing HIV Post-Exposure Prophylaxis, Testing, and New Diagnoses in Two Australian Cities with Different Lockdown Measures during the COVID-19 Pandemic

**DOI:** 10.3390/ijerph182010814

**Published:** 2021-10-14

**Authors:** Eric P. F. Chow, Jason J. Ong, Basil Donovan, Rosalind Foster, Tiffany R. Phillips, Anna McNulty, Christopher K. Fairley

**Affiliations:** 1 Melbourne Sexual Health Centre, Alfred Health, Melbourne, VIC 3053, Australia; Jason.ong@monash.edu (J.J.O.); TPhillips@mshc.org.au (T.R.P.); cfairley@mshc.org.au (C.K.F.); 2 Central Clinical School, Faculty of Medicine, Nursing and Health Sciences, Monash University, Melbourne, VIC 3004, Australia; 3 Centre for Epidemiology and Biostatistics, Melbourne School of Population and Global Health, The University of Melbourne, Melbourne, VIC 3052, Australia; 4 The Kirby Institute, The University of New South Wales, Sydney, NSW 2052, Australia; Bdonovan@kirby.unsw.edu.au (B.D.); Rosalind.Foster@health.nsw.gov.au (R.F.); 5 Sydney Sexual Health Centre, Sydney Hospital, Sydney, NSW 2000, Australia; Anna.Mcnulty@health.nsw.gov.au; 6 School of Public Health and Community Medicine, The University of New South Wales, Sydney, NSW 2052, Australia

**Keywords:** HIV, prevention, test, diagnosis, screening, COVID-19, coronavirus, transmission, sex, lockdown, SARS-CoV-2

## Abstract

Australia introduced a national lockdown on 22 March 2020 in response to the COVID-19 pandemic. Melbourne, but not Sydney, had a second COVID-19 lockdown between July and October 2020. We compared the number of HIV post-exposure prophylaxis (PEP) prescriptions, HIV tests, and new HIV diagnoses during these lockdown periods. The three outcomes in 2020 were compared to 2019 using incidence rate ratio. There was a 37% and 46% reduction in PEP prescriptions in Melbourne and Sydney, respectively, with a larger reduction during lockdown (68% and 57% reductions in Melbourne’s first and second lockdown, 60% reduction in Sydney’s lockdown). There was a 41% and 32% reduction in HIV tests in Melbourne and Sydney, respectively, with a larger reduction during lockdown (57% and 61% reductions in Melbourne’s first and second lockdowns, 58% reduction in Sydney’s lockdown). There was a 44% and 47% reduction in new HIV diagnoses in Melbourne and Sydney, respectively, but no significant reductions during lockdown. The reduction in PEP prescriptions, HIV tests, and new HIV diagnoses during the lockdown periods could be due to the reduction in the number of sexual partners during that period. It could also result in more HIV transmission due to substantial reductions in HIV prevention measures during COVID-19 lockdowns.

## 1. Introduction

During the COVID-19 pandemic, many countries implemented restrictions including stay-at-home orders, curfews, and social distancing rules. These restrictions have led to reductions in the number of sexual partners, asymptomatic HIV and sexually transmissible infection (STI) screening, and STI diagnoses during the pandemic [1,2,3,4,5,6].

In Australia, the first COVID-19 case was reported on 25 January 2020, and the number of COVID-19 cases has gradually increased thereafter [7]. In response to the COVID-19 pandemic, Australia closed its borders on 20 March 2020. In addition to the national response, each State Government also introduced different lockdown measures throughout 2020 based on the number of COVID-19 cases in each State and Territory [8,9]. Furthermore, in late March 2020, Victoria and New South Wales (i.e., the two most populous states) introduced social-distancing rules, stay-at-home orders, and closure of non-essential businesses. These restrictions were eased in early May 2020. However, a second and larger wave of COVID-19 emerged in Victoria in June 2020. Between 20 July and 2 August 2020, Victoria recorded 5914 COVID-19 cases, with only 207 cases reported by other jurisdictions in the same period [10]. In response to the second wave of COVID-19, Victoria underwent a 112-day lockdown between July and October 2020, which was stricter than the first lockdown, including a curfew (8 p.m.–5 a.m.) and a 5 km travel limit [10]. There have been no additional restrictions for people who are immunocompromised during the COVID-19 pandemic in Australia, but they have been advised to stay at home as much as possible [11]. There were no restrictions in other Australian states during the second lockdown in Victoria. Given Victoria had two lockdowns and New South Wales (NSW) only had one, it is hypothesised that the impact on HIV testing and diagnoses may be different between the two capital cities, Melbourne and Sydney.

This study aimed to examine and compare the effect of lockdown on PEP prescriptions, HIV testing, and HIV diagnoses at the largest sexual health clinics in these cities.

## 2. Materials and Methods

The Melbourne Sexual Health Centre (MSHC) and Sydney Sexual Health Centre (SSHC) are the largest public HIV/STI clinics in Victoria and New South Wales, respectively. Both clinics provide free HIV and STI testing and treatment. The MSHC diagnosed about 54% of new HIV cases and prescribed about 68% of PEP for the state of Victoria, while SSHC diagnosed about 10% of new HIV cases for the state of New South Wales. Both MSHC and SSHC remained open throughout 2020. In 2020, there was no change in medical staffing in both clinics; however, up to 10% at SSHC and 25% at MSHC of the nursing staff were redeployed to COVID-19 duties elsewhere (e.g., contact tracing) due to reductions in clinical loads at both clinics.

Both clinics use electronic medical records. We extracted data for the three outcome variables: (1) number of HIV tests, (2) new HIV diagnoses, and (3) HIV post-exposure prophylaxis (PEP) prescriptions at both clinics between 2019 and 2020. We used data from 2019 as the reference year to examine the changes before and during the COVID-19 pandemic. Furthermore, we extracted the number of COVID-19 cases from the Victorian and New South Wales Departments of Health [12,13].

The annual number of the three outcome variables was calculated. We reported the weekly number of HIV tests and PEP prescriptions, but not the number of new HIV diagnoses due to the small number of cases. The weekly number of HIV tests and PEP prescriptions during the lockdown periods in 2020 was calculated and compared with the corresponding weeks in 2019. The data from MSHC were also stratified into periods according to the lockdown periods in Melbourne in 2020: (1) pre-lockdown, from 1 January (week 1) to 21 March (week 12); (2) first lockdown, from 22 March (week 13) to 9 May (week 19); (3) post-first lockdown, from 10 May (week 20) to 4 July (week 27); (4) second lockdown, from 5 July (week 28) to 24 October (week 43); (5) post-second lockdown, from 25 October (week 44) to 31 December (week 53). Given there was no second lockdown in Sydney, we used the same period for pre-lockdown and first lockdown as per Melbourne, but the post-first lockdown period in Sydney was defined as the period from 10 May (week 20) to 31 December (week 53). Poisson regression coefficients were calculated. The incidence rate ratios (IRRs) for the three outcome variables were calculated by exponentiating the Poisson regression coefficients, and the corresponding 95% confidence intervals (CI) were calculated. We reported the IRR for the annual number of PEP prescriptions, HIV tests, and new HIV diagnoses in 2020 compared to 2019. Furthermore, we also reported the IRR for the number of PEP prescriptions and HIV tests for each lockdown period in 2020 compared to 2019.

All statistical analyses were conducted in Stata (version 17, StataCorp LP, College Station, TX, USA). This study was approved by the Alfred Hospital Ethics Committee (301/20) and the South Eastern Sydney Local Health District Human Research Ethics Committee (2021/ETH00428) with a waiver for informed consent for the use of routinely collected clinical data.

## 3. Results

### 3.1. Melbourne

There were 20,375 COVID-19 cases notified in Victoria in 2020, with the first wave hitting a peak of 489 cases during week 13 (22–28 March 2020), and the second wave hitting a peak of 3265 cases during week 31 (26 July to 1 August 2020).

**Figure 1:**
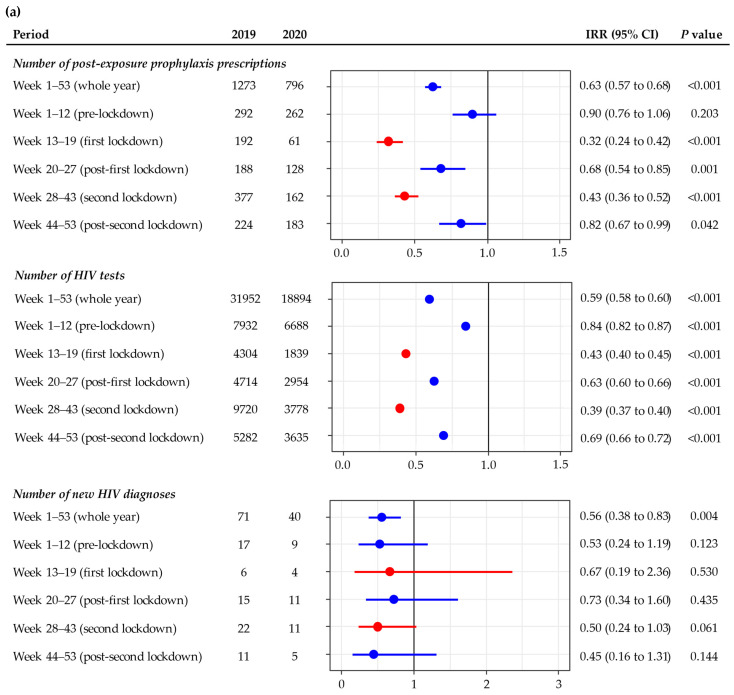

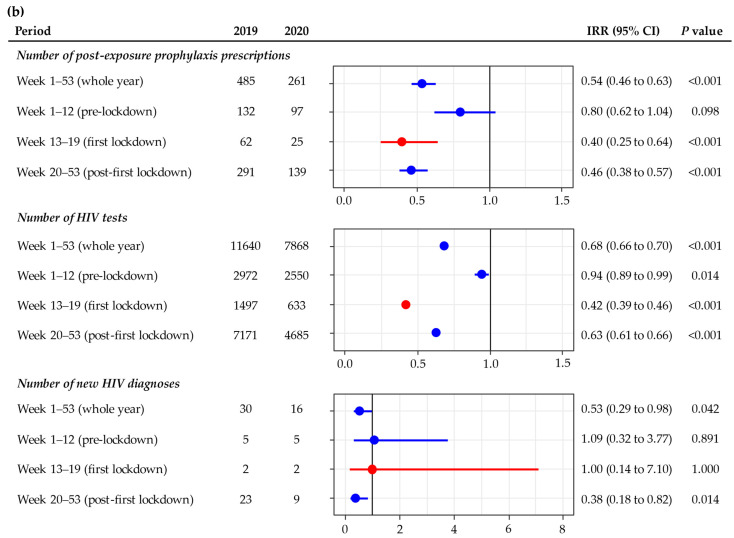
Incidence rate ratio of the number of HIV post-exposure prophylaxis prescriptions, HIV tests, new HIV diagnoses in 2020 compared to 2019 at the (**a**) Melbourne Sexual Health Centre and (**b**) Sydney Sexual Health Centre. The red dots and lines represent the lockdown periods.

**Figure 2:**
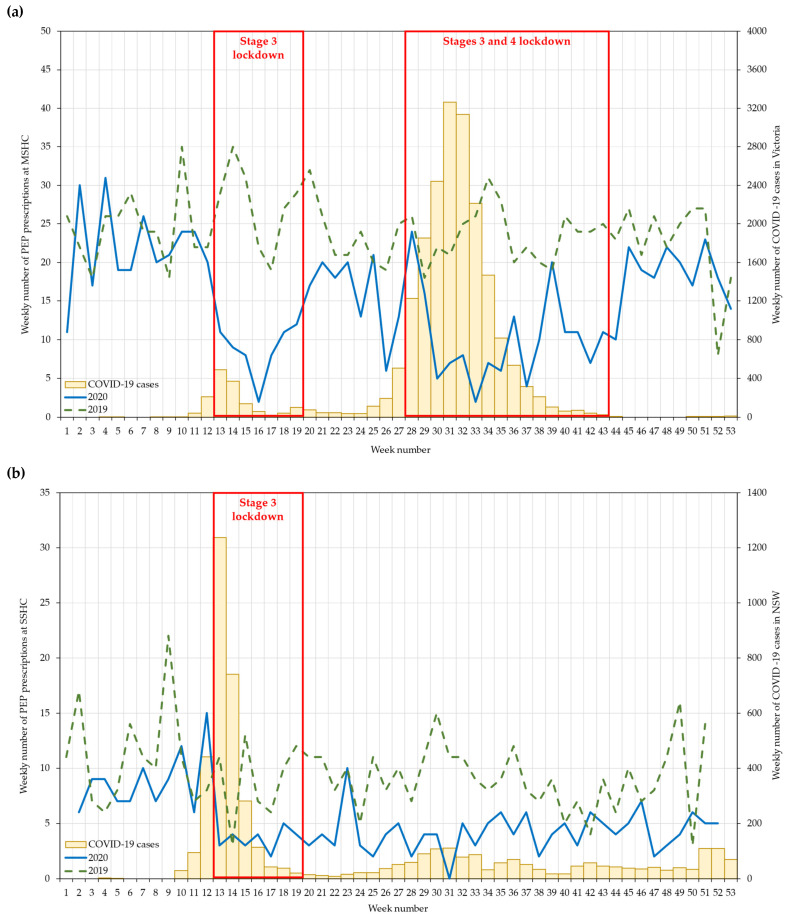
Weekly number of HIV post-exposure prophylaxis prescriptions at the (**a**) Melbourne Sexual Health Centre and (**b**) Sydney Sexual Health Centre, in 2019–2020. Data were not available in 2019 weeks 52 and 53, and 2020 weeks 1 and 53 at the Sydney Sexual Health Centre due to closure over Christmas and New Year. Stage 3 lockdown included social-distancing rules, stay-at-home orders, and closure of non-essential businesses. Stage 4 lockdown included Stage 3 lockdown measures in addition to a curfew (8 p.m.–5 a.m.) and a 5 km travel limit.

The annual number of PEP prescriptions reduced significantly from 1273 in 2019 to 796 in 2020 (IRR = 0.63; 95% CI: 0.57 to 0.68): the reduction affected all of 2020 except for the pre-lockdown period (week 1–12) (Figure 1a). The reduction in the number of HIV tests was more pronounced during the first lockdown (68%), followed by the second lockdown (57%). The weekly number of PEP prescriptions dropped dramatically in the first week of both lockdowns, and the number of PEP prescriptions gradually increased towards the end of each lockdown. The number of PEP prescriptions in the post-second lockdown period returned to a level similar in 2019 (Figure 2a).

**Figure 3:**
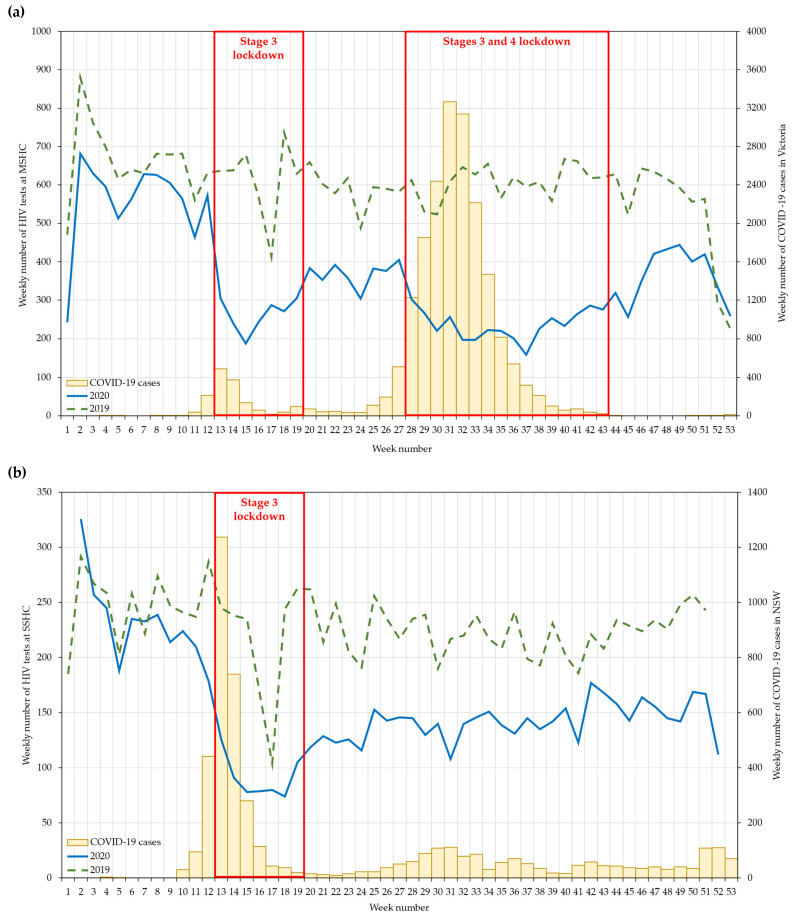
Weekly number of HIV tests at the (**a**) Melbourne Sexual Health Centre and (**b**) Sydney Sexual Health Centre, in 2019–2020. Data were not available in 2019 weeks 52 and 53, and 2020 weeks 1 and 53 at the Sydney Sexual Health Centre due to closure over Christmas and New Year. Stage 3 lockdown included social-distancing rules, stay-at-home orders, and closure of non-essential businesses. Stage 4 lockdown included Stage 3 lockdown measures in addition to a curfew (8 p.m.–5 a.m.) and a 5 km travel limit.

The annual number of HIV tests reduced significantly from 31,952 in 2019 to 18,894 in 2020 (IRR = 0.59; 95% CI: 0.58 to 0.60), and the reduction was significant in all periods (Figure 1a). The reduction in the number of HIV tests was more pronounced during the second (61%) than in the first lockdown (57%). The weekly number of HIV tests dropped dramatically in the first week of both lockdowns (week 13 and week 28, respectively): this number increased gradually four weeks after the start of the first lockdown but took about 14 weeks to increase after the start of the second lockdown. The number of HIV tests in post-second lockdown remained low, but it reached 2019 levels in the last two weeks of 2020 (Figure 3a).

The annual number of new HIV diagnoses reduced significantly from 71 in 2019 to 40 in 2020 (IRR = 0.56; 95% CI: 0.38 to 0.83), but this did not appear to relate to lockdown (Figure 1a).

### 3.2. Sydney

There were 4742 COVID-19 cases notified in NSW in 2020. The weekly number of COVID-19 cases increased gradually after March, hitting a peak of 1237 cases during week 13 (22–28 March 2020). Thereafter, it remained fairly stable, hovering at around 50–100 cases per week.

The annual number of PEP prescriptions reduced significantly from 485 in 2019 to 261 in 2020 (IRR = 0.54; 95% CI: 0.46 to 0.63), and the reduction was significant in the lockdown (60%) and post-lockdown (54%) periods, but not in the pre-lockdown period (Figure 1b). The number of PEP prescriptions remained at about five per week in the post-lockdown period (Figure 2b).

The annual number of HIV tests reduced significantly from 11,640 in 2019 to 7868 in 2020 (IRR = 0.68; 95% CI: 0.66 to 0.70), and the reduction was statistically significant in the pre- (6%), during (58%), and post-lockdown (37%) periods (Figure 1b). The weekly number of HIV tests dropped dramatically in the first week of lockdown (week 13); this number increased gradually towards the end of lockdown. The number of HIV tests during the post-lockdown period remained fairly stable, hovering at around 150 tests per week, but it was still lower than the number in 2019 (Figure 3b).

The annual number of new HIV diagnoses reduced significantly from 30 in 2019 to 16 in 2020 (IRR = 0.53; 95% CI: 0.29 to 0.98) (Figure 1b). There was a 62% reduction (from 23 to 9 cases) in the number of new HIV diagnoses in the post-lockdown period in 2020 when compared to 2019, but there was no change in the number of new HIV diagnoses in the pre-lockdown and lockdown periods.

## 4. Discussion

We found a large reduction in HIV PEP prescriptions and HIV tests in 2020, when compared to 2019, in Melbourne and Sydney. These reductions occurred during the lockdown period but HIV PEP prescriptions did not begin to rise substantially until there had been considerable reductions in COVID-19 cases, suggesting that it was the COVID-19 cases, as much as the lockdowns that were responsible for these declines. In Victoria, we found a larger reduction in HIV tests in the second lockdown than in the first lockdown, but this was not the case for PEP prescriptions. HIV diagnoses were lower in 2020 than in 2019 in both cities, and their percentage reduction reflected the reduction in the number of HIV tests. These findings suggest that substantial reductions in effective HIV prevention measures occurred due to the COVID-19 pandemic and the associated lockdowns: this could result in substantial undiagnosed HIV infections circulating in the community and, therefore, potentially being transmitted.

There was almost a 50% reduction in PEP prescriptions in 2020 when compared to 2019 at both clinics, with a larger reduction during the lockdown periods. Our results are consistent with international studies in Spain and the UK [14]. Similar to Australia, both Spain and the UK introduced a national lockdown around mid-March 2020. Sánchez-Rubio et al. examined PEP prescriptions from 20 hospitals in Madrid, and they found that there was a 37% reduction in PEP prescriptions between January and May 2020 when compared to 2019, but there was a 78% reduction during the lockdown period [14]. Similarly, Junejo et al. also reported an 82% reduction in PEP prescriptions four weeks before and after the lockdown in 2020 at 56 Dean Street in London [15]. In contrast, one of the largest HIV/STI clinics in Beirut, Lebanon, reported that there was no reduction but a 34% increase in PEP prescriptions between January and June 2020 when compared to 2019, with a three-month national lockdown from March to June 2020 [16]. These studies suggested some individuals still engaged in sexual risk-taking during the COVID-19 lockdown periods in different settings. Furthermore, our data provided an additional temporal trend, showing a rapid and sharp reduction in PEP prescriptions after the introduction of lockdown. PEP prescriptions recovered quickly after lockdown and returned to the pre-lockdown level, suggesting individuals resumed sexual risk-taking quickly once the restrictions and lockdowns were lifted.

The number of HIV tests significantly reduced in 2020 when compared to 2019, with a 41% reduction in Melbourne and a 32% reduction in Sydney, and the largest reductions were seen during the lockdown periods in both cities. The magnitude of reductions in HIV testing in Australia is similar to other countries. The Global Fund to Fight AIDS, Tuberculosis and Malaria reported a 41% reduction in HIV testing between April and September 2020 from 502 health facilities across 32 countries in Africa and Asia [17]. Consistent with our data, the largest reductions in these African and Asian countries occurred in March and April 2020 at the beginning of the COVID-19 pandemic and lockdown, but the number of HIV tests gradually increased thereafter [17]. Some individuals may have delayed their HIV testing during the peak of the COVID-19 pandemic in their settings, waiting for the situation to stabilise or for lockdown restrictions to be lifted [18]. Reductions in HIV testing during the COVID-19 pandemic may be due to the reduction in sexual risk [3,19], perception of low risk for HIV acquisition [20], and fear of acquiring COVID-19 when visiting a health service for HIV testing [18,20].

Apart from the individuals’ behavioural changes or attitudes, reductions in HIV testing may be due to disruptions in health services. The World Health Organization’s Global HIV, Hepatitis and STI Programmes survey revealed that 61 out of 144 countries (42%) reported disruptions in HIV/STI and hepatitis services between April and June 2020, and 38 out of 61 countries (62%) reported disruptions in HIV testing during the same period [21]. These disruptions may be due to the different lockdown measures and restrictions in different settings, including the closure of public transport, restrictions on private vehicles’ movement, closure of sexual health services, and suspension of non-emergency services during the lockdown periods, which may have impacted individuals access to HIV testing [18,22,23,24]. However, the reductions in HIV testing in our study were likely due to the changes in individuals’ behaviours or attitudes instead of structural disruptions, because Australian sexual health clinics remained open and provided HIV/STI services during the lockdown periods [25]. As the COVID-19 pandemic continues, an alternative approach to scaling up HIV testing would be required. HIV self-testing has been approved and is available in many countries worldwide. A meta-analysis of 10 randomised control trials has concluded that HIV self-testing can increase the uptake of HIV testing by 1.5 times when compared to standard-of-care testing [26]. Scaling up and implementing HIV self-testing could potentially maintain adequate access to HIV testing to support the overburdened healthcare services during the COVID-19 pandemic [27,28,29,30]. A survey of 685 Chinese men who have sex with men (MSM) revealed no change in the overall HIV testing number three months before and during the COVID-19 lockdown starting mid-January 2020 in China, but there was a significant shift from using facility-based HIV testing to HIV self-testing [31]. A similar observation was also seen in three Kenyan counties, where, in April 2020, there were more reports of HIV self-testing than there were of standard-of-care testing [32].

The number of HIV diagnoses was reduced by almost half in 2020 when compared to 2019 in both cities. Although the number of new HIV diagnoses reduced during the lockdown periods in 2020 when compared to 2019, these reductions were not statistically significant. There are several possible explanations. First, the small number of new HIV diagnoses in these periods may have limited the statistical power. Second, the number of individuals at risk of HIV has reduced due to the reduction in sexual risk-taking during the COVID-19 pandemic [19]. The HIV epidemic has changed during the 2010s [33]. Before the COVID-19 pandemic, new HIV diagnoses among Australian-born MSM were decreasing but new HIV diagnoses among overseas-born MSM were increasing, particularly individuals who are recently arrived migrants and international students [33,34,35,36]. Given Australia has closed its border to international travellers, fewer migrants and international students would have been tested for HIV in 2020 than in 2019 [37].

There are several limitations. First, this study was conducted in two major urban sexual health clinics, which may not be generalisable to the whole country. Second, this was a cross-sectional study and therefore we are unable to prove the causal relationship between the COVID-19 pandemic and the reductions in HIV PEP prescriptions, testing and new diagnoses. Third, some individuals who had been to the clinics previously might have received their HIV tests or accessed PEP prescriptions elsewhere due to the travel radius limit during the second lockdown in Victoria, even though the travel radius limit did not apply to individuals who require medical care.

## 5. Conclusions

Timely HIV testing and treatment are critical for HIV prevention and control. There were significant reductions in PEP prescriptions, HIV testing, and HIV diagnoses in Melbourne and Sydney during the COVID-19 pandemic, with a larger reduction during the lockdown periods. These reductions are likely due to changes in an individuals’ health-care-seeking behaviour rather than disruptions in sexual health services [38]. Additionally, past studies have also shown that there was a reduction in pre-exposure prophylaxis (PrEP) use during lockdowns among Australian MSM [19,20,39]. Disruptions in HIV prevention and treatment during the COVID-19 pandemic, even short-term disruptions, could jeopardise progress toward HIV elimination and could also lead to an increase in mortality rates [40,41,42]. Adequate access to emergency services such as PEP prescriptions is critical for HIV prevention during the COVID-19 pandemic. HIV self-testing could be a potential alternative approach to scale up HIV testing during and after lockdowns to maintain the HIV testing rate.

## Data Availability

Data is contained within the article.
